# Highly Deformable Porous Electromagnetic Wave Absorber Based on Ethylene–Propylene–Diene Monomer/Multiwall Carbon Nanotube Nanocomposites

**DOI:** 10.3390/polym12040858

**Published:** 2020-04-08

**Authors:** Hasti Bizhani, Ali Asghar Katbab, Emil Lopez-Hernandez, Jose Miguel Miranda, Raquel Verdejo

**Affiliations:** 1 Department of Polymer and Color Engineering, Amirkabir University of Technology, Hafez Ave., Tehran 1591634311, Iran; bijani@aut.ac.ir; 2 Institute of Polymer Science and Technology (ICTP-CSIC), C/ Juan de la Cierva 3, 28006 Madrid, Spain; emil.lopez@ictp.csic.es; 3 Department of Estructura de la Materia, Facultad de Físicas, Universidad Complutense de Madrid, 28040 Madrid, Spain; miranda@ucm.es

**Keywords:** EPDM, ethylene–propylene–diene monomer, multiwall carbon nanotubes, foams, electromagnetic interference, EMI shielding

## Abstract

The need for electromagnetic interference (EMI) shields has risen over the years as the result of our digitally and highly connected lifestyle. This work reports on the development of one such shield based on vulcanized rubber foams. Nanocomposites of ethylene–propylene–diene monomer (EPDM) rubber and multiwall carbon nanotubes (MWCNTs) were prepared via hot compression molding using a chemical blowing agent as foaming agent. MWCNTs accelerated the cure and led to high shear-thinning behavior, indicative of the formation of a 3D interconnected physical network. Foamed nanocomposites exhibited lower electrical percolation threshold than their solid counterparts. Above percolation, foamed nanocomposites displayed EMI absorption values of 28–45 dB in the frequency range of the X-band. The total EMI shielding efficiency of the foams was insignificantly affected by repeated bending with high recovery behavior. Our results highlight the potential of cross-linked EPDM/MWCNT foams as a lightweight EM wave absorber with high flexibility and deformability.

## 1. Introduction

The need for electromagnetic interference (EMI) shields has risen over the years as the result of our digitally and highly connected lifestyle, which will intensify with the advent of 5G and driverless cars, among others. EMI is defined as the adverse effects of electromagnetic emissions, such as auroras and cellular phones, among many others, on the performance of other devices [1,2]._ENREF_1 Hence, over the past five decades, scientists have optimized circuits and designed advanced materials to minimize EMI [3].

Conductive polymer composites, in particular, carbon-based filled systems, have already shown to be a lightweight solution for EM absorbing shields [4]. Among the initial studies, Yang et al. [5] developed polystyrene foams obtaining shielding efficiencies (SE) around 20 dB with 15 wt.% and 7 wt.% carbon nanofibers and nanotubes, respectively. Subsequent works improved the SE or decreased the loading fractions required to achieve commercially attractive EMI shielding materials (around 20 dB in the X-band region (8.2–12.4 GHz)) and mostly looked at thermoplastic or rigid thermoset matrices or coated the foam surfaces with conductive nanofillers [6,7,8,9,10,11,12,13,14,15,16,17,18,19,20]. These previous studies have demonstrated that the development of the cellular structure causes the redistribution of the nanoparticles, decreasing the average gap between the nanoparticles along the cell walls, thus enhancing the electrical conductivity and EMI shielding properties [9,14]. However, repeated loading may reduce the conductivity of these strategies as the coating spalls off the foam surface or point contacts within the rigid polymer loading are lost. Additionally, the scalability of some of the proposed methodologies is also limited. Hence, the use of rubber matrices could potentially reduce these shortcomings. Some studies have also reported adequate EMI shielding features of rubber nanocomposite foams, in particular, natural rubber (NR) filled with multiwall carbon nanotubes (MWCNTs) [21] and silicone rubber filled with MWCNTs/ferriferous oxide (Fe_3_O_4_) [22]. Zhan et al. fabricated closed-cell NR nanocomposite foams using supercritical CO_2_ and observed improved electrical, EMI shielding, and compressive properties [21]. Yang et al. investigated the effect of MWCNTs/Fe_3_O_4_ and reported average EM absorptions of up to 64% in the X-band [22].

Hence, the aim of this study was to develop an intrinsically conductive elastomeric foam with appropriate EMI shielding behavior that could withstand repeated bending using a scalable process. To that end, ethylene–propylene–diene monomer (EPDM) rubber, a copolymer of ethylene (45–75%), propylene monomers (13–45%), and nonconjugated dienes (ethylidene norbornene, dicyclopentadiene, and 1,4-hexadiene; 1–11%), was selected for its high hydrophobic characteristics as well as remarkable ozone and light resistivity [23]. These characteristics make EPDM the most commonly used rubber in the automotive and construction industries. In this study, we report on the fabrication of both solid and cellular EPDM/MWCNT nanocomposites by means of an industrial processing technology using an open two-roll mill and compression molding protocols. The materials developed here are highly flexible and deformable and present high EM absorbing characteristics, which are not significantly affected by repeated bending. 

## 2. Experimental

### 2.1. Materials

EPDM rubber, Keltan 2750 (ethylene content: 48 wt.%; 5-ethylidene-2-norbornene (ENB) content: 7.8 wt.%) with Mooney viscosity of ML (1 + 4) 100 °C = 28, was kindly provided by ARLANXEO (Geleen, Netherlands). Maleic anhydride grafted EPDM (EPDM-*g*-MA), Royaltuf^®^ 498, with Mooney viscosity of 30 (ML (1 + 4) 100 °C), maleic anhydride content = 0.8–1.2 wt.%, and density = 0.87 g/cm^3^, was purchased from Crompton—Uniroyal Chemical Co. (Connecticut, USA). 4,4′-Oxy-bis(benzenesulfonyl hydrazide) (OBSH, GENITRON^®^ OB) as an inorganic blowing agent was also provided by Lanxess (Leverkusen, Germany). MWCNTs, NC7000, were purchased from Nanocyl SA (Sambreville, Belgium) (average diameter, length, and surface area of 9.5 nm, 1.5 μm, and 250–300 m^2^/gr, respectively).

### 2.2. Compounding and Sample Preparation

All EPDM/MWCNT compounds were prepared according to ASTM D3182 using an open two-roll laboratory mill at 70 °C. EPDM rubber was first masticated and preheated for 5 min and then blended with EPDM-*g*-MA. Afterwards, dried MWCNTs were incorporated into the rubber band, followed by the sequential addition of the curing ingredients (zinc oxide (ZnO), stearic acid (SA), sulfur (S), and dicumyl peroxide (DCP)) and blowing agent (for foams, OBSH) for 20 min. The optimum rubber recipe (Table 1) was selected after studying the foaming behavior and morphology of a set of samples, varying the OBSH concentration. Milled compounds were left for 24 h and then compression molded using an electrically heated hydraulic press (Gumix) at 160 °C and 200 bars to optimum cure time (t_90_) of each individual compound. Both crosslinking and foaming occurred simultaneously during molding. 

### 2.3. Characterization

Basic characterization regarding curing, crosslink density, viscoelastic behavior, and thermal degradation of the compounds can be found in the Supplementary Materials. 

The density (ρ) was calculated directly from the mass–geometric volume relationship, ρ = m/V. The porosity percentage of foams was also calculated as 100(1 − ρ_f_/ρ_s_), where ρ_f_ and ρ_s_ denote the density of the foam and solid nanocomposite, respectively. 

Cryofractured cross sections of the samples were analyzed by scanning electron microscopy (SEM), Philips ESEM XL30 (Philips, Amsterdam, Netherlands), after being sputter-coated with Au/Pd. SEM images were processed with Digimizer software to calculate the cell density as N = (nM^2^/A)^1.5^, where n, M, and A are the cell number, magnification factor, and area, respectively. 

Uniaxial tensile tests were carried out at room temperature at a cross-head speed of 200 mm/min using an Instron 3366 universal testing machine (Instron Corp., Norwood, USA) (BS ISO 37). Compressive strength was evaluated on circular specimens, 28.6 ± 0.1 mm in diameter and 12.5 ± 0.5 mm in thickness, following ASTM D575-9, at a cross-head speed of 12 mm/min to 50% of their initial height. Both tensile and compression tests were carried out on a minimum of five specimens of each sample. 

Thermal conductivity tests were performed in accordance with ASTM E 1530 using LaserComp FOX 50 (TA Instruments, Wakefield, USA) on cylindrical specimens, 51 mm diameter and 12.5 mm thickness, with an experimental error of less than 5% of the absolute value. 

Complex dielectric permittivity (ε* = ε′ − jε′′) measurements were performed using ALPHA high-resolution Novocontrol broadband dielectric spectrometer (Novocontrol, Aachen, Germany) from 0.1 Hz to 10 MHz at room temperature. The samples were fixed in the cell between two parallel gold-plated electrodes (the diameters of upper and lower electrodes were 20 and 30 mm, respectively). The amplitude of the applied AC electric signal was 1 V. 

The EMI shielding efficiency and extent of EM wave absorption were analyzed using an Agilent E8364B PNA vector network analyzer (Keysight Technologies Inc., Santa Rosa, USA) within the frequency range of the X-band. The measurements were performed on rectangular samples, 22.9 × 10.2 mm^2^ and 3 ± 0.2 mm in thickness. The reported values of the dielectric permittivity and EMI SE tests are the averages of five samples for each compound.

## 3. Results and Discussion

**Figure 1:**
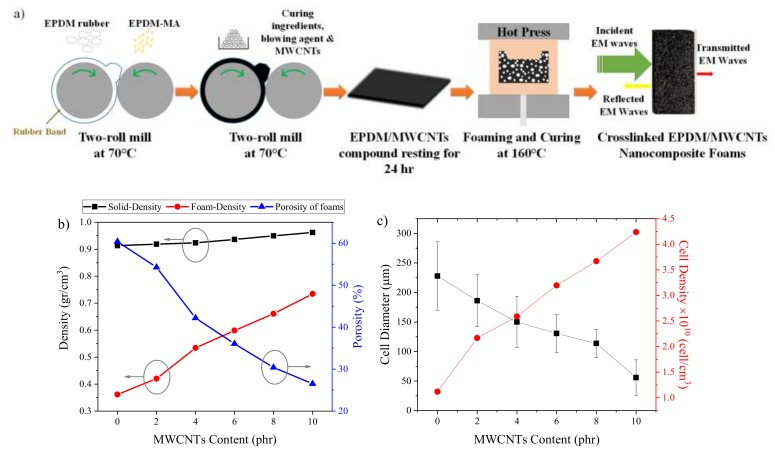
(**a**) Schematic representation of the sample preparation. (**b**) Density of the samples and porosity of foams as a function of multiwall carbon nanotubes (MWCNT) content. (**c**) Cell diameter and cell density of the foams as a function of MWCNT content.

We selected an extensively used rubber processing technology to prepare the EPDM nanocomposites based on an open two-roll mill, for mastication and mixing of the rubber ingredients, followed by compression molding, for the vulcanization and foaming (Figure 1a). Rubber foams are obtained through the decomposition reaction of a chemical agent, OBSH in this case, and occurs simultaneously with the vulcanization reaction. Thus, the kinetics of these reactions have to be well adjusted to obtain a stable system; i.e., if the vulcanization is too slow, the foam collapses, and if it is too fast, the foam does not fully expand. We studied these reactions, analyzing the cure curve (Figure S1), the cross-link density (CLD) (Table S1), and the viscoelastic characteristics of the materials (Figure S2). A detailed explanation of these results is given in the Supplementary Materials. In summary, MWCNTs affect the reaction kinetics by activating the vulcanization reaction and forming a reinforcing 3D physical network within the EPDM matrix. Additionally, the foaming agent acts as an accelerator due to the presence of sulfohydrazide and its by-products, which could activate the sulfur vulcanization of EPDM [24,25,26].

The densities of the solid and foam samples (Figure 1b) increased as a function of MWCNT content. The density increase of the solid samples is related to the higher intrinsic density of the MWCNTs; meanwhile, the density increase of the foams is the result of the observed differences in foaming and vulcanization kinetics. The mentioned differences, i.e., the acceleration of the vulcanization reaction and the increase in both CLD and melt viscosity of the compounds, limited the growth of the cells (Figure 1c). Similar results have been reported on reactive nanocomposite foams, where the kinetics of the reactions are also critical [11,27,28]. 

**Figure 2:**
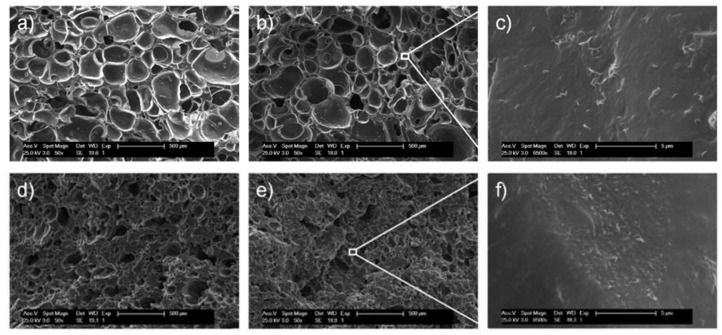
SEM micrographs of cryofractured ethylene–propylene–diene monomer (EPDM)/MWCNT nanocomposite foams containing (**a**) 0, (**b**) 2, (**d**) 6, and (**e**) 10 phr MWCNTs. (**c**,**f**) Micrographs of the MWCNTs within the morphology.

These differences are clearly apparent in the cellular morphology (Figure 2), with a cell diameter decrease from 227 ± 58 μm down to 59 ± 30 μm for unfilled and 10 phr MWCNT samples, respectively. Moreover, MWCNTs act as nucleating sites during the foaming process, which leads to the increase in cell number and, hence, the observed decrease in cell size. Yu et al. [29] have reported similar results in ethylene vinyl acetate (EVA)/MWCNT nanocomposite foams developed via chemical blowing agent. SEM micrographs of solid nanocomposites (Figure S3) clearly show the different fracture mechanisms of the samples: from the classical brittle fracture of the unfilled sample to the rough and wavy character of the filled samples, which is a further indication of the reinforcing effect of the MWCNTs. This reinforcing character is ascribed to a good interfacial interaction between the EPDM matrix and the MWCNTs resulting from the presence of the EPDM-*g*-MA [30].

Table 2 and Figure S4 show the mechanical properties of the samples under both tensile and compression. As expected from the reinforcing effect mentioned above, MWCNTs gradually increased the value of tensile and compression strength at several deformations (50%, 100%, 300%, and 500% for tensile and 10%, 30%, and 50% for compression), while the elongation at break for the solid samples decreased. Tensile strength at 50% strain increased up to 151% and 568% for solid and foam samples with 10 phr MWCNTs, respectively, while in compression, the increases were up to 60% and 1267% for solid and foam, respectively. This larger improvement of the MWCNTs in the elongation at break of the foamed samples is attributed to the decrease in the porosity. Dindarloo et al. prepared similar EPDM foams containing 2 phr MWCNTs, observing similar trends in the elongation at break and stress [31]. It is worth mentioning that the sample prepared in this study showed higher improvement for tensile properties, which could be assigned to a better interfacial adhesion and state of dispersion of the MWCNTs due to the EPDM-*g*-MA. 

Thermal conductivity can be a valuable asset in EMI shielding applications, both to improve EM wave absorption via heat dissipation and to minimize the temperature impact on the electronic components. Stationary measurements showed thermal conductivity variations from 0.185 W/(m·K) to 0.267 W/(m·K) for the unfilled and 10 phr MWCNTs in the solid materials, respectively, and from 0.069 to 0.206 W/(m·K) for their foamed counterparts, respectively (Figure S5). The obtained value is higher compared to the reported values for most of the foamed nanocomposites prepared for EMI application [11,14,32,33]. Hence, MWCNTs increased the thermal conductivity of the EPDM up to nearly 45% and 200% for the solid and foamed samples, respectively. The modest improvement of the solid samples, compared to the increase in orders of magnitude in electrical conductivity, has previously been observed and ascribed to the mismatch in the phonon spectra of the two phases [34,35]. Meanwhile, the thermal conductivity of the foam samples occurred through three main mechanisms: conduction, convection, and radiation. Both convection and radiation in these samples can be considered negligible as the cell sizes were below 4 mm [36] and the relative density was above 0.2 [37]. Thus, the main mechanism was conduction through the solid and gas phases. Hence, the observed improvement in the thermal conductivity was a combination of the improved thermal conductivity through the matrix and the decrease in porosity. This increase in the thermal conductivity of the sample did not affect the onset of the thermal degradation (Figure S6). 

**Figure 3:**
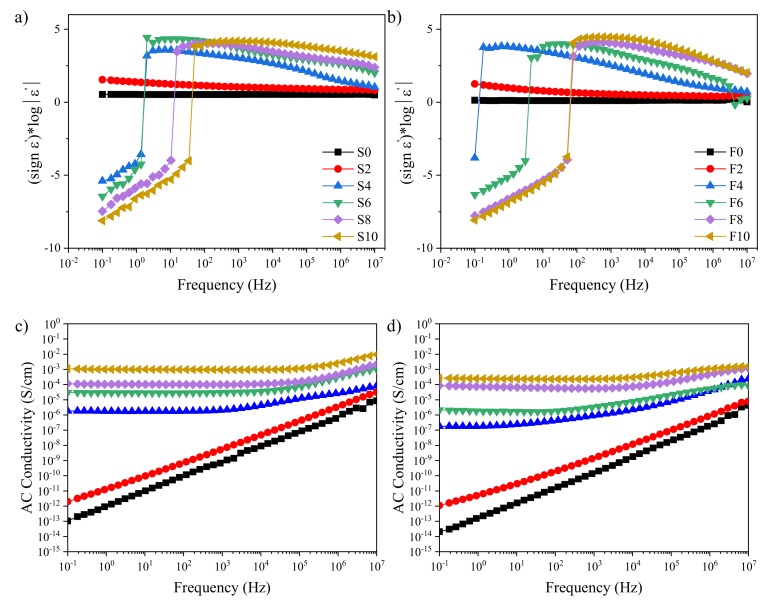
Logarithmic plots of ε’ (**a**,**b**) and AC conductivity (**c**,**d**) as a function of frequency for solid (left) and foam (right) samples.

Complex dielectric permittivity is defined as ε* = ε′ − jε′′, where ε’ and ε’’ denote the real and imaginary parts of the permittivity, respectively. The ε’ represents the electric energy storage capacity of a material when subjected to an external field. Meanwhile, the ε’’ represents its ability to dissipate the electric field energy as heat through dielectric polarization processes. Figure 3 shows the permittivity and AC conductivity behavior as a function of the frequency at room temperature. In the high-frequency region, we observed an increase of the ε′ with filler content for both solid and foamed samples. This could be attributed to the volumetric effect of the filler and enhanced interfacial charge accumulation [38]. Meanwhile, in the low-frequency region, samples with high loading fractions exhibited negative permittivity. Contrary to the high positive permittivity, which is mainly due to electron localization, a plasma resonance brought up by electron delocalization can create the negative permittivity. This phenomenon has been ascribed to the coexistence of the applied external electric field and an electric field generated by the delocalized electrons [39]. At low frequencies (f < f_p_, where f_p_ is the plasma resonance frequency), the oscillation rate of the electrons in the structure of the conductive component (in our case, the MWCNTs) becomes faster than the field leading to the negative permittivity [39,40]. At high frequencies (f > f_p_), electrons vibrate slower than the external field, and the real part of permittivity becomes positive. Among the different models that explain the dielectric behavior of materials, the Drude model is the one that can predict the negative permittivity behavior [41] and is considered as an indication of the formation and existence of a continuous conductive network throughout the matrix [39,40,42]. In our case, this negative ε’ was observed in samples with concentrations ≥ 4 phr MWCNTs. This result is consistent with the increase in melt storage modulus and nonterminal behavior of the nanocomposites with 4 phr or more reported in the SI. For these samples, the dominant dielectric loss (Figure S7) mechanism was conductance loss, which means that the electric charges transferred within the polymer matrix by direct contact between MWCNTs [10,38]. Further evidence of the existence of a percolated 3D network is the frequency-independent behavior of the AC conductivity at low frequencies, with an increase in conductivity values from 10^−13^ up to 10^−3^ S/cm. 

**Figure 4:**
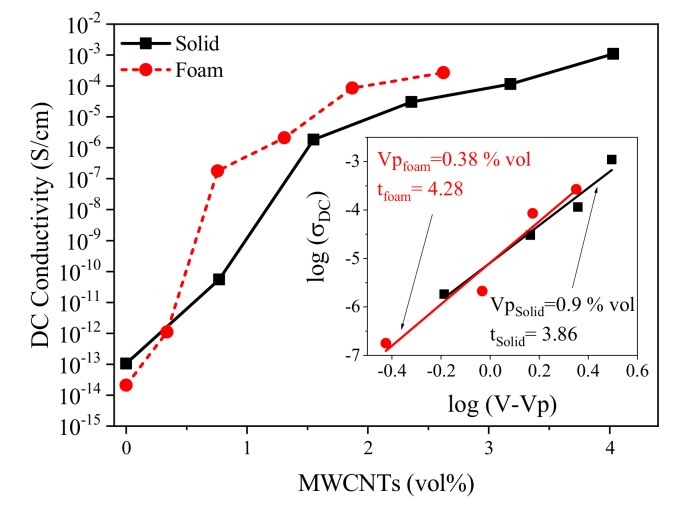
DC electrical conductivity of solid and foamed nanocomposites as a function of filler content. The inset shows the log–log plot of DC conductivity versus (V − V_p_).

In order to study the influence of foaming on the electrical conductivity, the DC electrical conductivity (σ_DC_) of the nanocomposites was calculated from the AC data (σ_AC_) as [43]

(1) $$\sigma_{\mathrm{AC}}=\sigma_{\mathrm{DC}}+{A\omega }^{s}$$

where ω = 2πf; σ_DC_ is the DC electrical conductivity, which is originated from ionic or electronic conductivity; A is a constant; and *s* is a low power exponent. For polymers, s = 1, and the σ_DC_ value is governed by the σ_AC_ at low frequency. Figure 4 shows the σ_DC_ as a function of MWCNT volume fraction, where a typical electrical percolation behavior is observed with a sudden increase of the conductivity values. We subsequently calculated the concentration of the percolation threshold (Figure 4, inset) by fitting the curve to a power law equation $\sigma_{\mathrm{DC}}=\sigma_{0}{\left(V-V_{p}\right)}^{t}$, where σ_0_ is a scaling factor related to the filler intrinsic conductivity, V is the nanofiller volume fraction, V_p_ is the volume fraction at the percolation threshold, and t is a critical exponent. The results showed that the foams outperformed the solid samples, presenting higher conductivities and lower percolation threshold, 0.4 vol % compared to 0.9 vol %. This obtained percolation threshold is in a suitable range compared to other conductive nanocomposite foams [7,9,33,44]. This behavior has previously been ascribed to a decrease in the interparticle distances between the MWCNTs and to an increase of the MWCNT contacts and orientation due to the biaxial extensional stretching during the cells’ growth [9,10,19].

**Figure 5:**
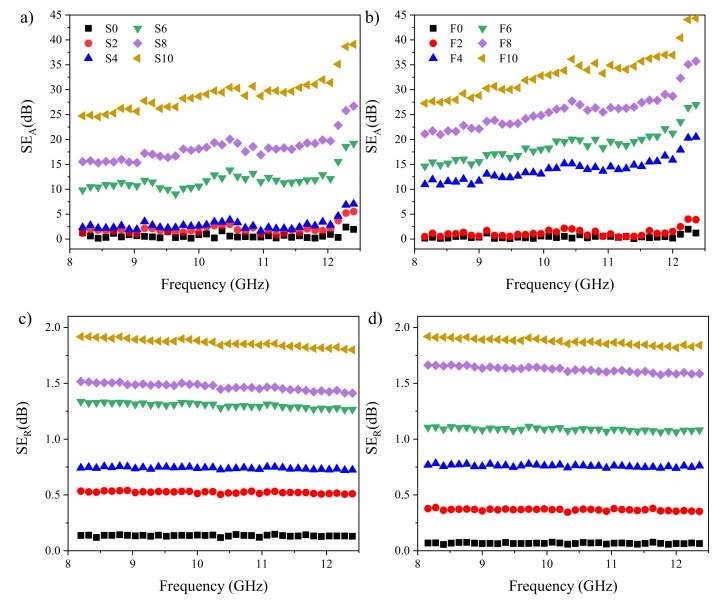
SE_A_ (**a**,**b**) and SE_R_ (**c**,**d**) of solid (left) and foam (right) samples in the X-band frequencies.

**Figure 6:**
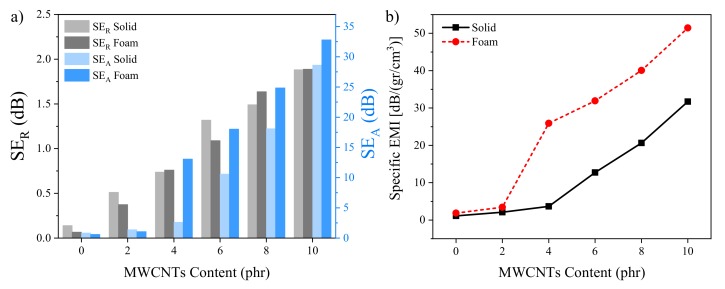
(**a**) SE_A_ and SE_R_ and (**b**) specific electromagnetic interference (EMI) shielding efficiency (SE) of the samples at 10 GHz.

EMI shielding can occur through three mechanisms: (1) reflection from the surface of the shield (SE_R_) due to the impedance mismatch between the free space and the shield, (2) absorption as the wave goes through the shield (SE_A_), and (3) multiple reflection (SE_M_), which can happen due to the heterogeneity within the material (cell and nanoparticles). Hence, according to Schelkunoff [45], the overall shielding efficiency of a layer can be expressed as

(2) $$\mathrm{SE}\left[\mathrm{dB}\right]={\mathrm{SE}}_{A}\left[\mathrm{dB}\right]+{\mathrm{SE}}_{R}\left[\mathrm{dB}\right]+{\mathrm{SE}}_{M}\left[\mathrm{dB}\right]$$

(3) $${\mathrm{SE}}_{A}\left[\mathrm{dB}\right]=20\log\left|e^{-\gamma_{s}t}\right|$$

(4) $${\mathrm{SE}}_{R}\left[\mathrm{dB}\right]=20\log\left|\frac{{\left(1+k\right)}^{2}}{4k}\right|$$

(5) $${\mathrm{SE}}_{M}\left[\mathrm{dB}\right]=20\log\left|1-\frac{{\left(k-1\right)}^{2}}{{\left(k+1\right)}^{2}}e^{2\gamma_{s}t}\right|$$

where k is the ratio of the impedance of the free space (η_0_) to that of the shielding material (η_s_) and can be obtained by $k=\eta_{0}/\eta_{s}=\sqrt{\varepsilon_{r}/\mu_{r}}$. γ_s_ is the wave propagation constant in the shield material, expressed as [46]

(6) $$\gamma_{s}=i\omega \sqrt{\varepsilon_{r}\varepsilon_{0}\mu_{r}\mu_{0}}$$

where μ_0_ and ε_0_ are the permeability and permittivity of vacuum, respectively. It is worth noticing that, if the thickness of the shield is greater than the skin depth ($\delta =1/\sqrt{\pi \mu \sigma f}$), i.e., t > δ, SE_M_ approaches zero, which means the multiple reflection effect is negligible [16]. SE_A_ and SE_R_ (Figure 5) for the prepared solid and cellular EPDM/MWCNT nanocomposites with a thickness of 25 mm and different MWCNT contents were calculated based on the measured permittivity and permeability in the X-band frequency. The permittivity results are shown in Figure S8. Due to the absence of magnetic filler, the real and imaginary parts of permeability were almost equal to 1 and 0, respectively, within the X-band and are not presented here. The unfilled samples were almost transparent to the EM radiations over the X-band. Meanwhile, the addition of MWCNTs gradually increased SE_A_ and SE_R_ with nanofiller content for both solid and foam nanocomposites. This result is ascribed to the increase in both electrical conductivity and high specific surface area of the MWCNTs. We also observed that the rate of increase of SE_A_ was higher than that of SE_R_, indicating that the dominant shielding mechanism was the absorption of the EM wave (Figure 6a). The SE percentages of each mechanism are reported in Table S2.

It is worth mentioning that, above the percolation threshold (≥ 4 phr), foamed samples exhibited higher SE_A_ compared to their solid counterparts (Figure 6a). Meanwhile, below the percolation threshold, no significant differences were observed between the solid and foam samples [33]. Thus, although the key factor governing the EMI SE is the conductive network, the observed change of the cellular structure, i.e., decreased cell size and increased cell density, should also affect it. This change appeared to increase the phase heterogeneity, thus increasing attenuation of the wave energy through multiple reflections. Foam samples displayed higher total SE than their solid counterparts, with the foamed materials with 6 phr of MWCNTs reaching 19.1 dB compared to 11.9 dB, at 10 GHz. Hence, this sample was close to the target value for commercial applications (20 dB). Meanwhile, the samples filled with 10 phr MWCNTs showed excellent EMI SE performance, ~30 and 35 dB at 10 GHz for solid and foam samples, respectively. The excellent electrical conductivities derived from electron tunneling, hopping, and conduction are closely linked to the ohmic loss, which is regarded as a crucial part of microwave absorption [47,48]._ENREF_53 Moreover, in the case of the foams, the scattering effects within the cellular structural increased the extent of the wave energy dissipation and, hence, increased the total EMI shielding values. Finally, we normalized the total EMI SE by the density, specific EMI SE, to properly compare the SE of the solid and foam samples (Figure 6b). This calculation clearly showed the excellent shielding efficiency of the foams compared to the solid samples. 

**Figure 7:**
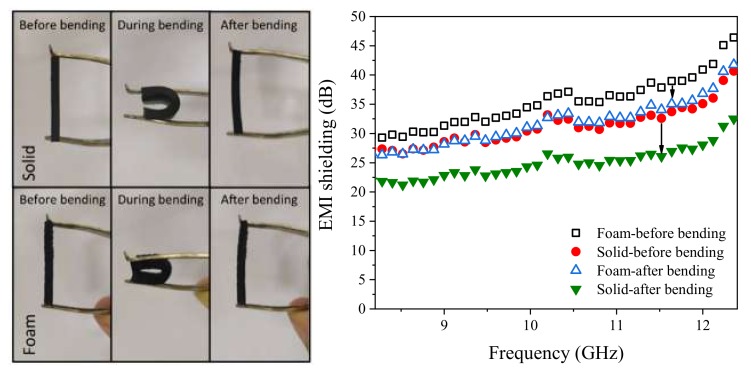
Photos (**left**) and EMI SE (**right**) of the samples filled with 10 phr MWCNT before and after repeated bending.

The inadequate flexibility of many conventionally used EMI shielding materials is considered as one of the main shortcomings hindering their application. Moreover, the stability of the dielectric and EMI shielding behavior of shields subjected to consecutive strains is another serious concern of both manufactures and final users. Figure 7a shows the optical photos of the vulcanized solid and foamed EPDM/MWCNT nanocomposites loaded with 10 phr MWCNTs before and after bending. Both solid and foamed samples could be bent freely by applying a low force and, after removal of the force, the samples reversed back to their initial dimensions within a short time. However, the foamed nanocomposites exhibited faster elastic shape recovery compared to the solid counterpart as a result of the air molecules trapped in the cells. We then assessed the effect of repeated bending to a radius of 2 mm for 1000 times (Figure 7b) upon the EMI shielding behavior in these samples. Solid and foam samples exhibited a slight decrease of around 22.1% and 9%, respectively, in the total EMI SE over the X-band frequency range. This small decrease should be the result of a change in the interconnectivity of the 3D MWCNT network within the EPDM matrix. Such modification of the 3D network would be restricted in the foam due to the topological constraints to which MWCNTs are subjected within the cellular structure, and the decrease of EMI performance after repeated bending is therefore smaller.

Finally, the use of different parameters, such as type of filler/matrix, frequency of measurement, fabrication methods, filler concentrations, and thicknesses, hindered the direct comparison of the SE found in the literature on rubber foams. Nevertheless, we have compiled the data (Table 3) in order to provide a comprehensive analysis of the EMI SE of rubber foam nanocomposites. From this data, it can be seen that the developed EPDM/MWCNT foams exhibited superior specific EMI SE and deformability. In addition, properties such as resistance to chemicals, moisture, and ozone should be ensured due to the use of EPDM rubber.

## 4. Conclusions

In summary, the present work has developed an industrially scalable lightweight EMI shielding material based on EPDM rubber and MWCNTs. The developed foam samples exhibit high thermal conductivities up to 0.2 W/m·K, high electrical conductivities of up to 2.7 × 10^−4^ S/cm, and EMI shielding efficiencies of up 45 dB, which do not degrade significantly after repeated bending. Such properties are the result of the formation of a 3D interconnected network within the EPDM matrix. 

Even though the developed samples already display high potential as an EMI shield over X-band frequency, further studies could optimize the cellular structure at large loading fractions. This optimization could then result in improved shielding efficiencies. Nevertheless, the samples developed here are suitable for many applications, such as transport, cell phones, and lightweight portable devices. The results obtained highlight the high potential of cross-linked EPDM/MWCNT foams as a lightweight wave absorber with high flexibility and deformability. 

## Figures and Tables

**Table 1:** Formulation (in phr, parts per hundred of rubber) of the prepared nanocomposite compounds, where S stands for solid and F for foam.

	S0	S2	S4	S6	S8	S10	F0	F2	F4	F6	F8	F10
EPDM	100	100	100	100	100	100	100	100	100	100	100	100
EPDM-*g*-MA	5	5	5	5	5	5	5	5	5	5	5	5
MWCNTs	0	2	4	6	8	10	0	2	4	6	8	10
ZnO	5	5	5	5	5	5	5	5	5	5	5	5
STA	1.5	1.5	1.5	1.5	1.5	1.5	1.5	1.5	1.5	1.5	1.5	1.5
DCP	1	1	1	1	1	1	1	1	1	1	1	1
S	0.5	0.5	0.5	0.5	0.5	0.5	0.5	0.5	0.5	0.5	0.5	0.5
OBSH	0	0	0	0	0	0	5	5	5	5	5	5

**Table 2:** Mechanical properties of solid and foamed EPDM/MWCNT nanocomposites.

Sample	Tensile Stress (MPa)				Strain at Break (%)	Compressive Stress (MPa)		
	50%	100%	300%	500%		10%	30%	50%
S0	0.62 ± 0.04	0.87 ± 0.10	1.5 ± 0.1	2.1 ± 0.2	641 ± 53	0.35 ± 0.01	1.4 ± 0.02	3.6 ± 0.1
S2	0.74 ± 0.03	1.05 ± 0.03	1.9 ± 0.1	2.8 ± 0.3	630 ± 31	0.39 ± 0.02	1.5 ± 0.03	4.0 ± 0.2
S4	0.83 ± 0.05	1.19 ± 0.07	2.5 ± 0.1	3.6 ± 0.1	600 ± 39	0.52 ± 0.04	1.7 ± 0.06	4.2 ± 0.1
S6	1.05 ± 0.03	1.59 ± 0.05	3.6 ± 0.1	5.2 ± 0.2	560 ± 42	0.63 ± 0.01	2.0 ± 0.07	5.3 ± 0.1
S8	1.27 ± 0.06	1.98 ± 0.07	4.6 ± 0.1	6.6 ± 0.2	520 ± 39	0.64 ± 0.04	2.2 ± 0.1	5.5 ± 0.3
S10	1.56 ± 0.07	2.51 ± 0.09	5.9 ± 0.1	-	483 ± 28	0.72 ± 0.08	2.4 ± 0.1	5.8 ± 0.2
F0	0.15 ± 0.02	0.23 ± 0.03	0.5 ± 0.1	-	344 ± 40	0.04 ± 0.01	0.1 ± 0.01	0.22 ± 0.02
F2	0.29 ± 0.03	0.47 ± 0.05	1.0 ± 0.2	-	351 ± 21	0.06 ± 0.02	0.15 ± 0.02	0.33 ± 0.06
F4	0.50 ± 0.05	0.77 ± 0.07	1.5 ± 0.1	-	361 ± 75	0.11 ± 0.04	0.3 ± 0.1	0.70 ± 0.19
F6	0.70 ± 0.07	1.09 ± 0.07	2.3 ± 0.2	-	377 ± 54	0.18 ± 0.03	0.5 ± 0.1	1.25 ± 0.03
F8	0.91 ± 0.07	1.40 ± 0.09	3.2 ± 0.1	-	392 ± 65	0.25 ± 0.06	0.7 ± 0.1	1.94 ± 0.33
F10	0.97 ± 0.10	1.54 ± 0.14	3.5 ± 0.2	-	405 ± 37	0.33 ± 0.01	1.1 ± 0.1	2.94 ± 0.4

**Table 3:** EMI SE of rubber foam nanocomposites in the X-band frequency range.

Material	Foam Fabrication Procedure	Filler Content (wt.%)	EMI Shielding		Mechanical Properties	Other Characteristics	Ref.
			Specific EMI SE (dB/gr cm^-3^)	Thickness (mm)			
NR/MWCNTs	ScCO_2_	6.4	40	1.3	Compressive stress at 30%: 0.14 MPa	Segregated CNT network	[21]
PDMS/MWCNTs	ScCO_2_	10	72	2	N/A	Flexible-Retained EMI shielding properties after bending	[22]
EPDM/MWCNTs	OBSH foaming agent	10	52	25	Compressive stress at 30%: 1.06 MPa, tensile stress at 300%: 3.46 MPa, strain at break: 405%	Chemical, moisture, and ozone resistive-Retained EMI shielding properties after bending	**This work**

## Supplementary Materials

The following are available online at https://www.mdpi.com/2073-4360/12/4/858/s1. Figure S1: Curing rheographs of solid and foamed EPDM/MWCNT nanocomposite compounds at 160 °C. Figure S2: (a) Storage modulus, (b) complex visocisty versus angular frequency, and (c) the relaxation time spectra at different MWCNT concentrations. Figure S3: SEM images of cryofractured surfaces of EPDM/MWCNT nanocomposites containing (a) 0, (b) 2, (c) 4, (d) 6, (e) 8, and 10 phr MWCNTs. Figure S4: Representative stress–strain curves of the solid (top) and foam (bottom) samples. Tensile tests (left) and compressive (right). Figure S5: Thermal conductivity of solid and cellular EPDM/MWCNT nanocomposites versus MWCNT loading at room temperature. Figure S6: TGA curves of EPDM/MWCNT nanocomposites with different MWCNT contents: (a) solid and (b) foam. The corresponding 10% weight loss temperatures are shown in the insets. Figure S7: Logarithmic curves of the imaginary permittivity (ε″) vs. frequency for EPDM/MWCNT nanocomposites in the frequency region of 10^−1^ to 10^7^ Hz: (a) solid and b) foam. Figure S8: Dielectric properties of solid and foamed nanocomposites in the X-band frequencies: real (top) and imaginary (bottom) parts of the relative permittivity for solid samples (left), and real (top) and imaginary (right) parts of the relative permittivity for foamed samples (right). Table S1: Curing characteristics for solid and foamed EPDM/MWCNT nanocomposites. Table S2: SE percentages of each mechanism at 10 GHz.
