# Supplementary material for: Highly Deformable Porous Electromagnetic Wave Absorber Based on Ethylene–Propylene–Diene Monomer/Multiwall Carbon Nanotube Nanocomposites
Source: Polymers (Basel). 2020 Apr 8;12(4):858. doi: 10.3390/polym12040858 (PMC7240433; doi:10.3390/polym12040858)
Supplement: Supplementary file 1 [file polymers-12-00858-s001.pdf]

Supplementary information

Highly Deformable Porous Electromagnetic Wave Absorber Based on Ethylene-Propylene-Diene-Monomer/Multiwall Carbon Nanotube Nanocomposites

Hasti Bizhani[1], Ali Asghar Katbab[1,*], Emil Lopez-Hernandez[2], Jose Miguel Miranda[3] and Raquel Verdejo[2,*]

[1] Department of Polymer and Color Engineering, Amirkabir University of Technology, Hafez Ave., Tehran 1591634311, Iran

[2] Institute of Polymer Science and Technology (ICTP-CSIC), C/ Juan de la Cierva 3, 28006, Madrid, Spain

[3] Dpt. Estructura de la Materia, Facultad de Físicas, Universidad Complutense de Madrid, 28040 Madrid, Spain

* Corresponding authors: katbab@aut.ac.ir; rverdejo@ictp.csic.es

1. CURING BEHAVIOR AND CROSSLINK DENSITY (CLD)

Curing characteristics of the prepared compounds were evaluated at 160 °C according to the ASTM 5289 using a rubber curing rheometer (Rubber Process Analyzer, Alpha Technologies, Akron, USA). Scorch time (t_2) and optimum cure time (t_{90}) were measured based on the obtained rheographs.

Degree of crosslinking at t_{90} for each individual compound was evaluated by means of equilibrium swelling method using Flory–Rehner equation (Eq. S1) [1]. For this purpose, five circular cured test pieces with a diameter of 1 cm were cut and immersed in toluene (99.5%) at room temperature for 72 h [2].

$$\nu = -\frac{1}{V}\left[\frac{\ln(1-V_R)+V_R+\mu V_R^{\,2}}{V_R^{1/3}-\frac{V_R}{2}}\right] \qquad \text{Eq. S1}$$

where ν is the CLD (mol/ cm^3), V_R is the volume fraction of EPDM rubber after immersion in toluene, V is the molecular volume of toluene (106.8 cm^3/mol), and μ is the interaction parameter between EPDM and toluene (0.5).

Figure S1 shows the curing curves of all fabricated EPDM/MWCNTs nanocomposite compounds containing various levels of MWCNTs, with and without foaming agent obtained at 160°C. The values of minimum torque (M_L), maximum torque (M_H), scorch time (t_2), and t_{90} (optimum cure time, the time for the torque that is equal to the 90% of $\Delta M=M_H-M_L$), are also given in Table S1. The results reveal that ΔM values increase with increasing MWCNTs content, which is indicative of the reinforcing effect of the MWCNTs [3-5]. Additionally, the scorch time reduces, from about 5 min to around 1 min, indicating an acceleration of the start of the vulcanization reaction. This result could be attributed to the higher thermal conductivity of both solid and foamed EPDM/MWCNTs nanocomposites (thermal conductivities are reported below). In hot curing of rubber compounds, the speed of temperature rise in various points of the compound is governed by thermal diffusivity factor ($\alpha = \kappa / \rho.C_p$; where κ, ρ, and C_P denote thermal conductivity, mass density, and specific heat capacity, respectively). Hence, as κ increases, the time required for the compound to reach the mold temperature becomes shorter [3]. Finally, foamed samples also exhibit lower values of scorch time and t_{90} compared to the solid samples with the same MWCNTs contents, indicating an

acceleration effect of the blowing agent. The decomposition of sulphohydrazide and its byproducts activate the sulfur vulcanization of EPDM. Similar observation has also been reported by other researchers [3-6].

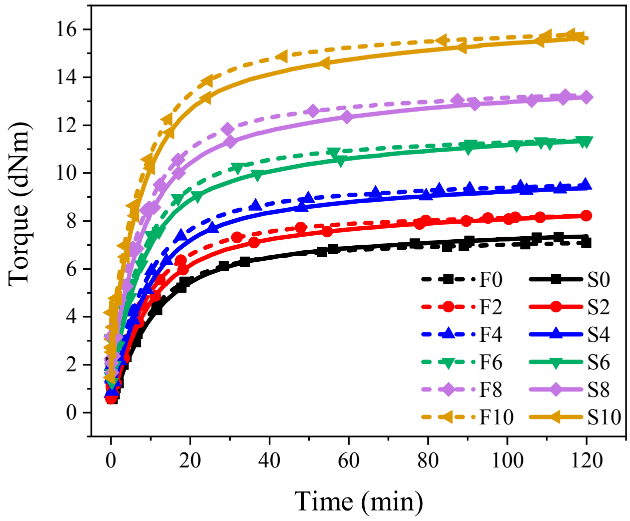

Figure S1. Curing rheographs of solid and foamed EPDM/MWCNTs nanocomposite compounds at 160°C.

Table S1. Curing characteristics for solid and foamed EPDM/MWCNTs nanocomposites.

Sample	Scorch time (min)	t_{90} (min)	M_H (dNm)	M_L (dNm)	ΔM (dNm)	M_c (g/mol)	$\nu \times 10^{-5}$ (mol/ cm^3)
S0	5.1	48.2	7.4	0.5	6.8	12270±440	4.1 ± 0.1
S2	4.3	50.1	8.2	0.7	7.5	10010±575	5.0 ± 0.3
S4	3.6	46.8	9.4	0.9	8.5	8903±426	5.6 ± 0.3
S6	2.5	49.3	11.4	1.3	10.1	7363±88	6.8 ± 0.1
S8	1.8	47.4	13.2	1.7	11.5	6654±160	7.5 ± 0.2
S10	1.5	45.3	15.6	2.5	13.2	5642±216	8.9 ± 0.3
F0	4.6	38.3	7.1	0.6	6.5	11660±1341	4.3 ± 0.5
F2	3.5	36.7	8.2	0.7	7.5	9922±479	5.0 ± 0.3
F4	2.9	37.4	9.5	0.9	8.6	9009±551	5.6 ± 0.3
F6	2.2	35.3	11.4	1.44	9.9	8235±690	6.1 ± 0.5
F8	1.5	35.1	13.3	1.87	11.4	6455±939	7.7 ± 1.1
F10	1.1	33.8	15.8	2.71	13.1	5643±296	8.9 ± 0.4

The CLD value would influence the mechanical properties such as deformation and elasticity as well as ease of segmental polarization when subjected to high-frequency electrical waves. Therefore, CLD of both solid and foamed samples at their corresponding optimum cure (t_{90}) was measured by the swelling method using the Flory–Rehner equation (Eq. S1). Table S1 shows the CLD of both solid and foamed samples increases with MWCNTs loading. This would be attributed to the activation of crosslinking reaction by MWCNTs particles, and also physical crosslinks formed by the MWCNTs within the microstructure of the samples [7-8]. It is worth to note that introducing cellular structure does not have any obvious effect on CLD. Moreover, the obtained results are in consistency with the results discussed earlier.

2. DYNAMIC MELT RHEOLOGICAL BEHAVIOR

The flowability of the compounds plays a crucial role in the foaming evolution and morphology of the cellular structure. Hence, we analyze the flow behavior and rheological properties as well as dispersion of MWCNTs, using dynamic oscillatory rheometry in the molten state by a reo-mechanical spectrometer (RMS, Paar Physica UDS 200, Graz, Austria). Oscillatory shear melt rheological measurements were conducted using a parallel-plate geometry with a diameter of 25 mm and a gap of 1 mm under controlled shear deformation mode. The linear viscoelastic characteristics of the samples without curing and foaming ingredients were recorded at 160 °C within the angular frequency range of 0.01–1000 (rad/s) with a small strain amplitude of 1% (in the linear viscoelastic region, which was obtained from the strain sweep test). Figure S2.a and S2.b show the variation of the storage modulus (G') and complex viscosity (η^*) versus frequency, respectively. The sample without MWCNTs exhibits Rouse-like viscoelastic behavior i.e. terminal behavior of G' at low angular frequency range; whereas all compounds containing MWCNTs show pseudo solid-like or nonterminal behavior, indicating the presence of physical networks of MWCNTs within the EPDM matrix. This is consistent with the increase in melt elasticity (G') and η^* as well as shear-thinning characteristics with MWCNTs loading fraction [9-10].

To get an insight into the micromorphology, extent of polymer-filler interaction, and dispersion state of MWCNTs, the linear viscoelastic behavior of the prepared nanocomposites was analyzed via calculation of relaxation time spectrum for storage and viscous modulus data by using Paar Physica UDS 200 software. Figure S2.c displays the weighted relaxation spectra, $\lambda.H(\lambda)$, versus λ, where $H(\lambda)$ and λ denote the relaxation time distribution function and relaxation time, respectively. Interestingly, the neat EPDM shows three characteristics peaks located at 0.0126 s, 0.13 s, and 1.34 s. These could be associated with the presence of micro-phases including, ethylene, propylene segments in the backbone of EPDM chain, and attached ethylene long branches with different ease of relaxation. Meanwhile, the inclusion of MWCNTs leads to a change in the weighted relaxation spectra. The low concentration sample (2 phr MWCNTs) presents only one broad peak, i.e. the chain motion is slowed down by the presence of the MWCNTs and their possible interaction with the micro-phases. Higher concentrations (>2 phr) exhibit no characteristic relaxation peak, typical of solid-like features, i.e. large relaxation times, and, hence, suggest the presence of 3D physical networks by the MWCNTs within the EPDM matrix. This is consistent with the dynamic melt rheological results presented in Figure S2.a and b, as MWCNTs particles percolate with each other and restrict the molecular motion leading to no or infinite relaxation times. Similar observation has been reported for the nanocomposites based on Poly(lactic acid) and cellulose nanocrystals [11].

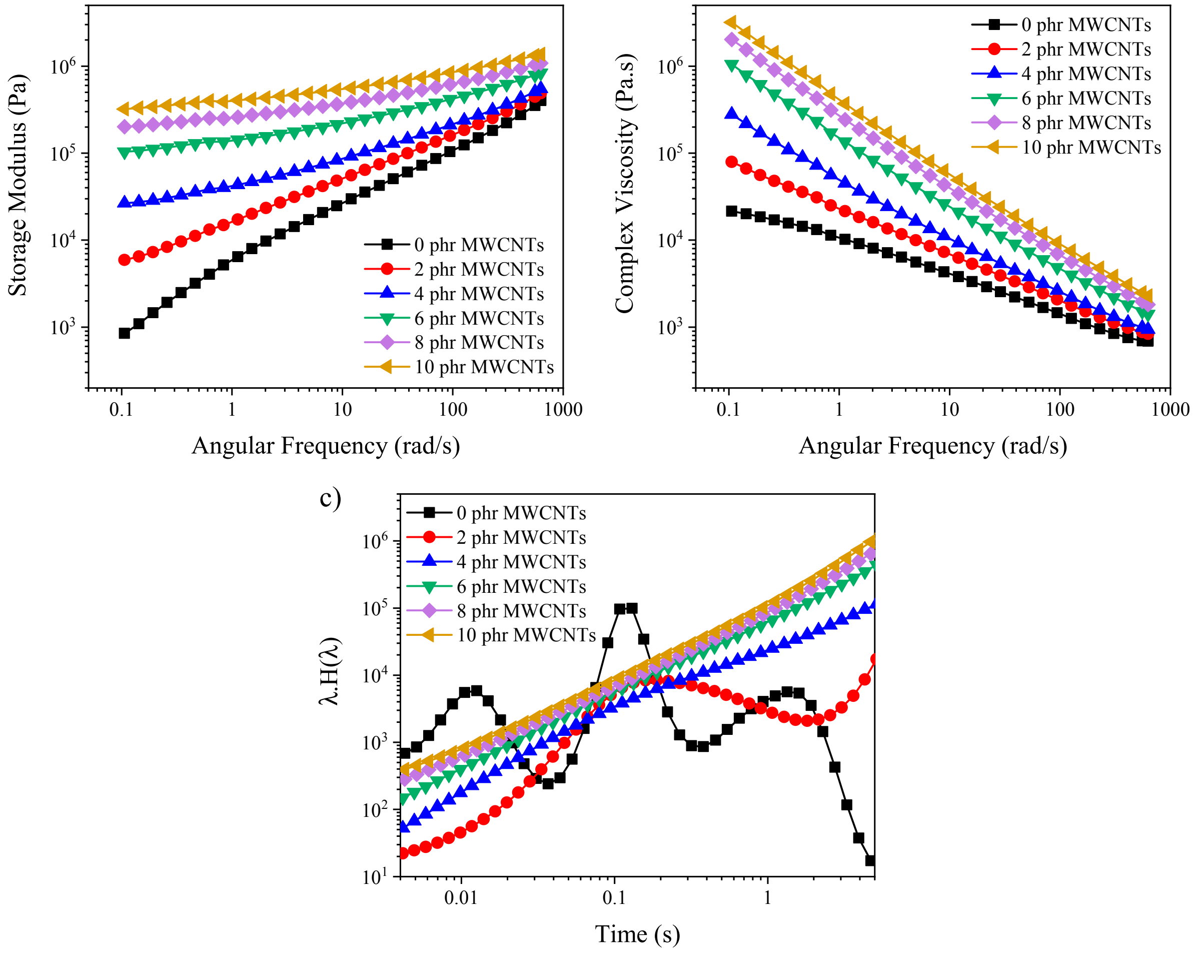

Figure S2. (**a**) Storage modulus and (**b**) complex visocisty versus angular frequency and (**c**) the relaxation time spectra at different MWCNTs concentrations.

3. MORPHOLOGY

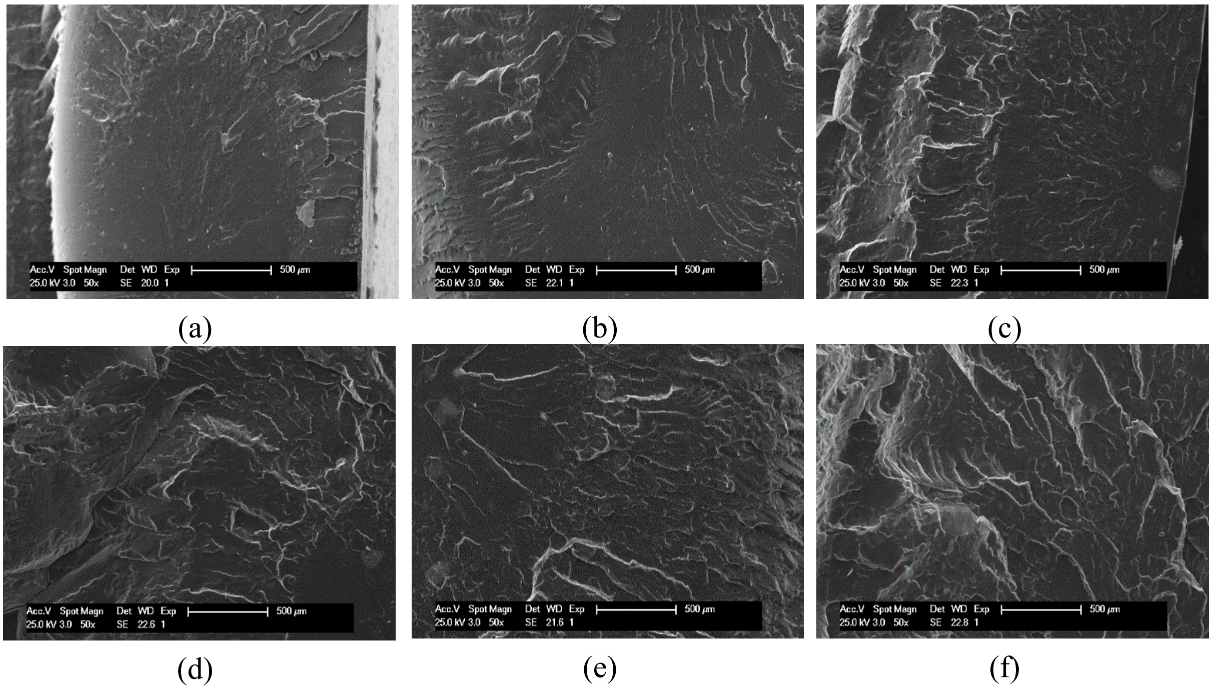

Figure S3. SEM images of cryo-fractured surfaces of EPDM/MWCNTs nanocomposites containing: (**a**) 0, (**b**) 2, (**c**) 4, (**d**) 6, (**e**) 8, and (**f**) 10 phr MWCNTs.

4. MECHANICAL PROPERTIES

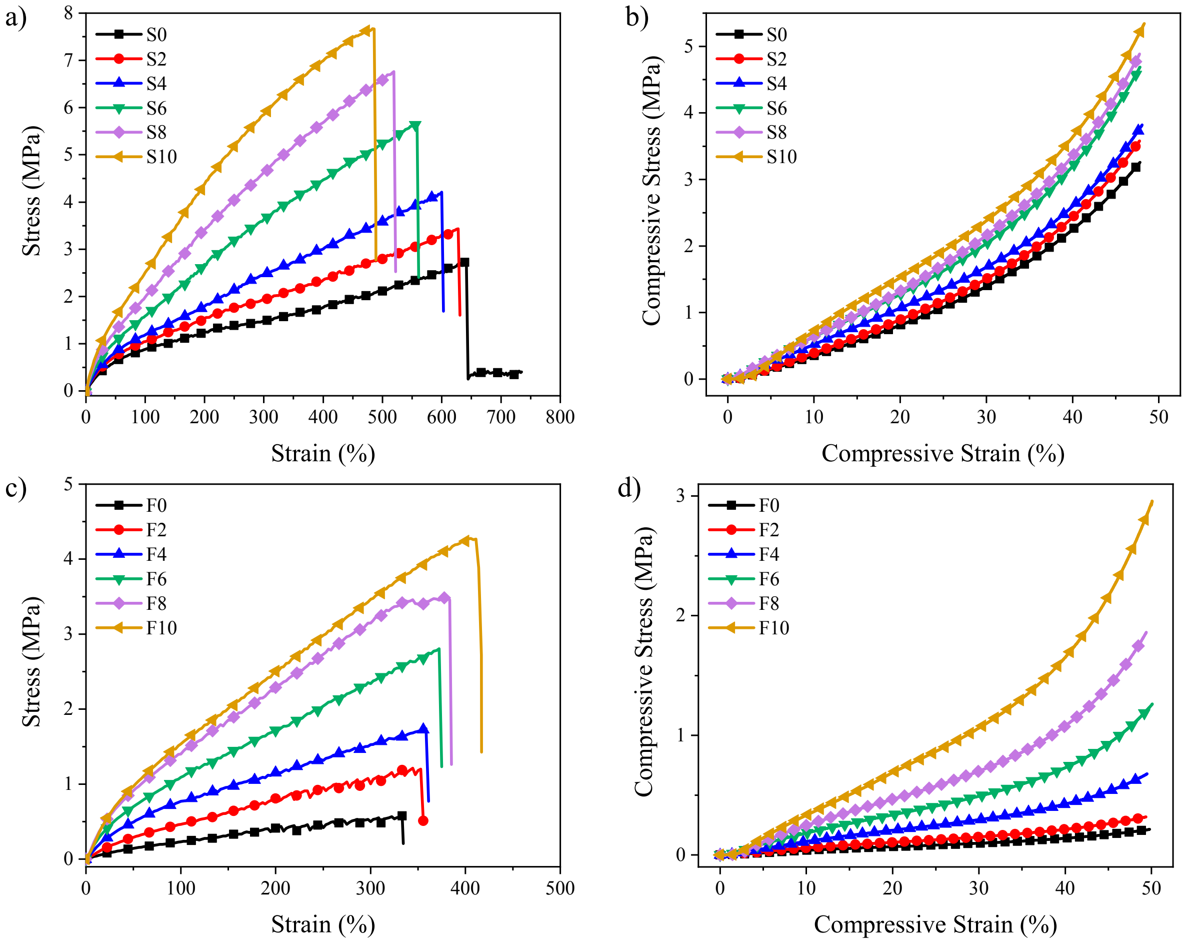

Figure S4. Representative stress-strain curves of the solid (**top**) and foam (**bottom**) samples. Tensile tests (**left**) and compressive (**right**).

5. THERMAL CONDUCTIVITY

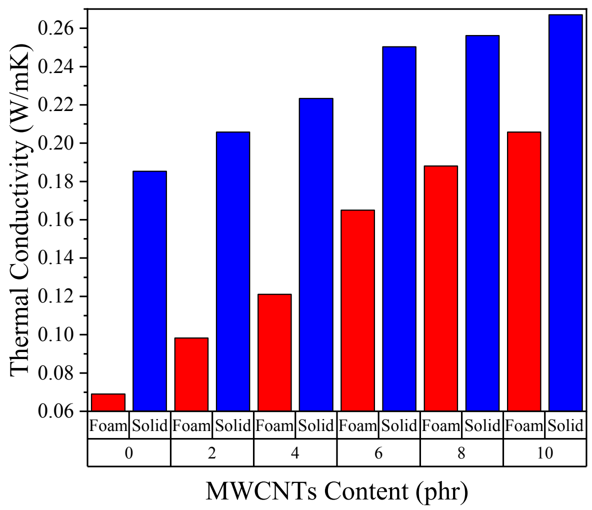

Figure S5. Thermal conductivity of solid and cellular EPDM/MWCNTs nanocomposites versus MWCNTs loading at room temperature.

6. THERMOGRAVIMETRIC ANALYSIS (TGA)

For better understanding, the extent of MWCNTs dispersion and interfacial adhesion between the EPDM matrix and MWCNTs, thermogravimetric analysis (TGA) was conducted on the prepared samples using a thermal analyzer, TA Instruments Series Q500 (New Castle, USA). Samples with the weight of 20 mg were heated from 40 to 800 °C at a heating rate of 10 °C/min under nitrogen atmosphere (with a flow rate of 90 mL /min).

As a high-performance polymer nanocomposite, thermal stability is considered as a key parameter for high-tech applications. The TGA thermographs of solid and foamed EPDM/MWCNTs nanocomposites are displayed in Figure S6.a and b, respectively. As can be seen, compared to the neat and unfilled EPDM samples all EPDM/MWCNTs nanocomposites exhibit higher temperatures for the onset of thermal degradation. This indicates that EPDM segments are thermally shielded by MWCNTs aggregates. However, the foamed samples display lower T_{90} (the temperature that the sample loses only 10% of its weight) which is attributed to the presence of air molecules inside the cells, activating the onset of thermal degradation of EPDM segments.

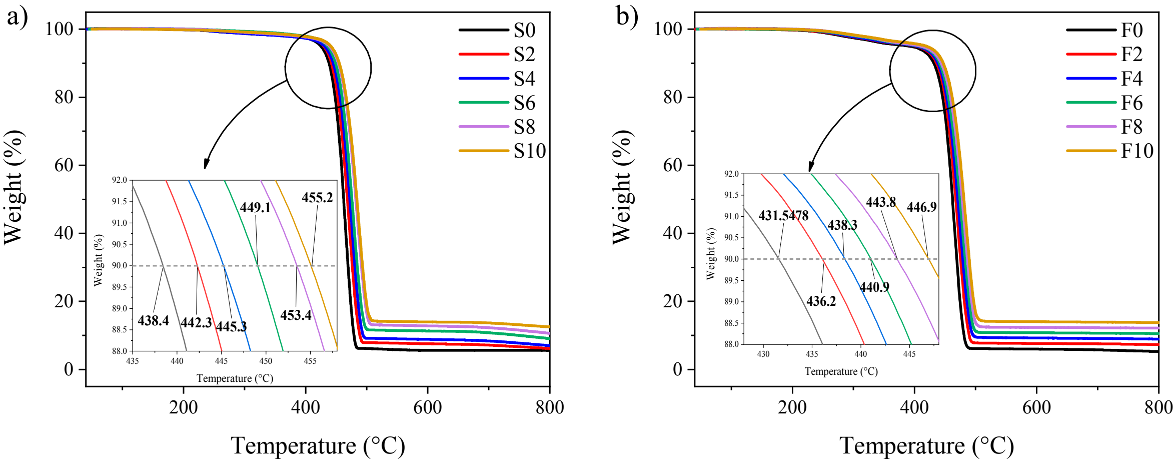

Figure S6. TGA curves of EPDM/MWCNTs nanocomposites with different MWCNTs contents (**a**) Solid; (**b**) Foam. The corresponding 10% weight loss temperatures are shown in the insets.

7. DIELECTRIC PERMITTIVITY

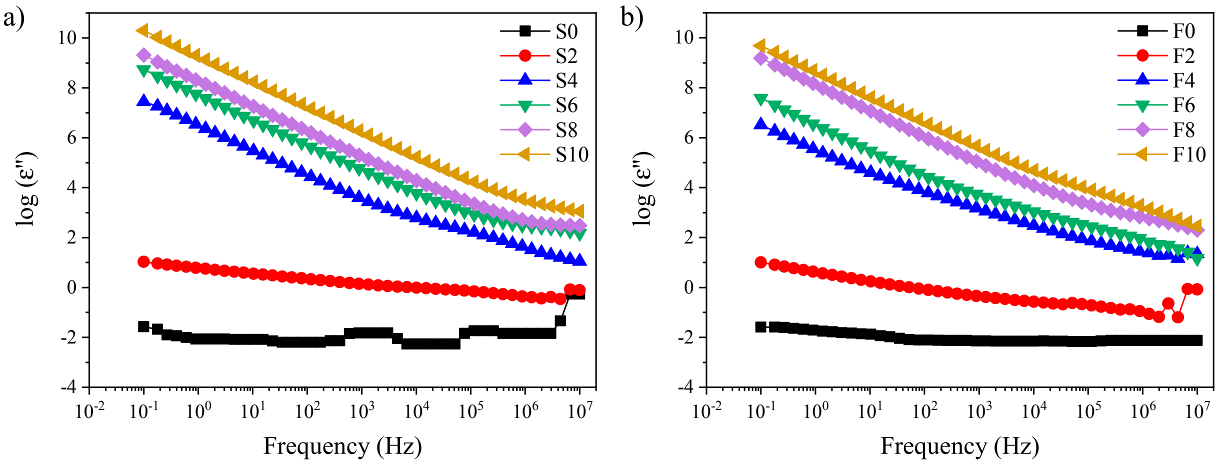

Figure S7. Logarithmic curves of the imaginary permittivity (ε'') vs. frequency for EPDM/MWCNTs nanocomposites in the frequency region of 10^{-1} to 10^{7} Hz: (**a**) Solid and (**b**) foam.

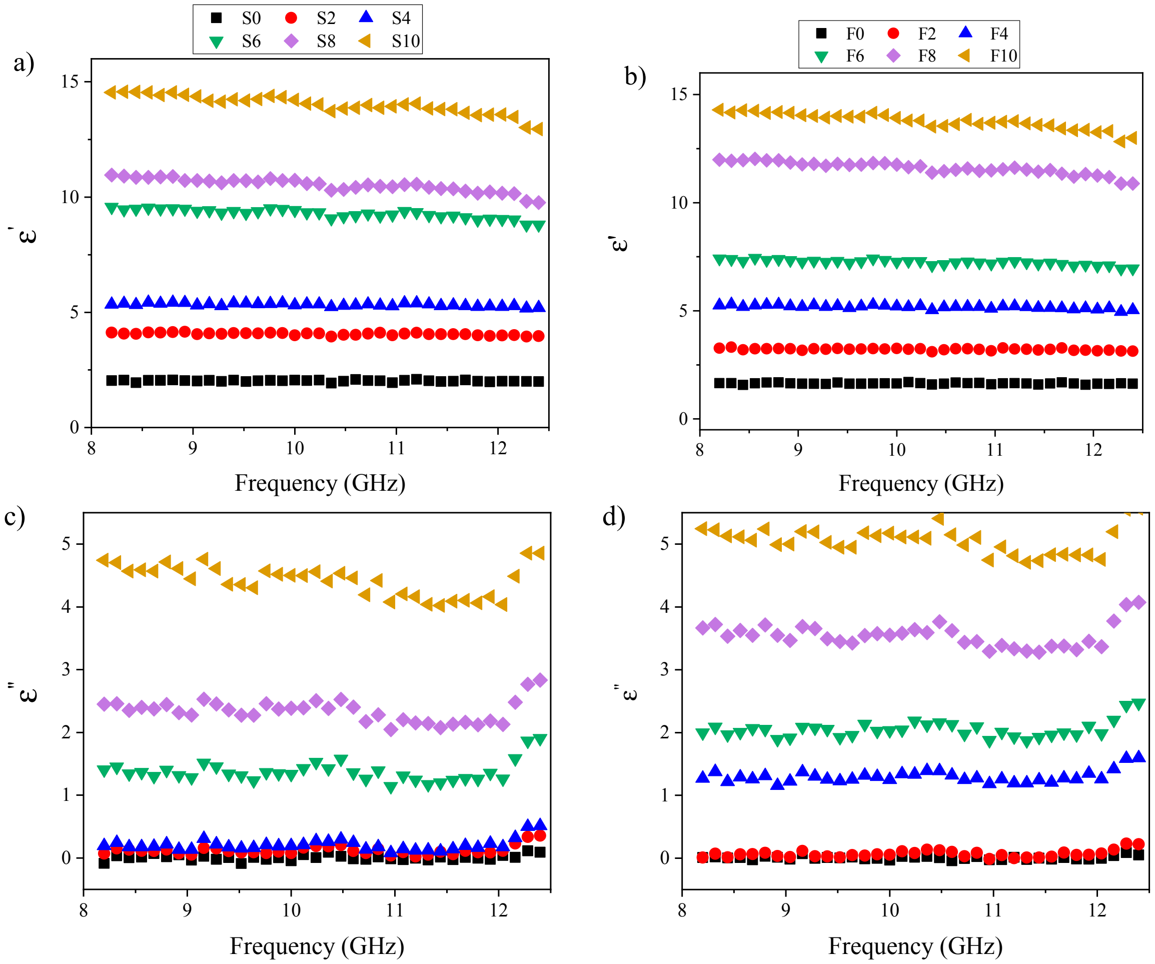

Figure S8. Dielectric properties of solid and foamed nanocomposites in the X-band frequencies: real (**top**) and imaginary (**bottom**) parts of the relative permittivity for solid samples (**left**), and real (**top**) and imaginary (**right**) parts of the relative permittivity for foamed samples (**right**).

Table S2. SE percentages of each mechanism at 10 GHz.

MWCNTs (phr)	Solid			Foam		
	T(%)	R(%)	A(%)	T(%)	R(%)	A(%)
0	79	3	17	86	2	13
2	64	11	25	72	8	20
4	46	16	38	4	16	80
6	6	26	67	1	22	77
8	1	29	70	0	31	68
10	0	35	65	0	35	65

T: Transmission. R: Reflection. A: Absorption.

REFERENCES

1. Flory, P. J.; Rehner Jr, J., Statistical mechanics of cross-linked polymer networks I. Rubberlike elasticity. *J. Chem. Phys.* **1943,** *11* (11), 512-520.
2. Su, J.; Chen, S.; Zhang, J.; Xu, Z., Comparison of cure, mechanical, electric properties of EPDM filled with Sm2O3 treated by different coupling agents. *Polym. Test.* **2009,** *28* (3), 235-242.
3. Nakaramontri, Y.; Kummerlöwe, C.; Nakason, C.; Vennemann, N., The effect of surface functionalization of carbon nanotubes on properties of natural rubber/carbon nanotube composites. *Polym. Compos.* **2015,** *36* (11), 2113-2122.
4. Lu, L.; Zhai, Y.; Zhang, Y.; Ong, C.; Guo, S., Reinforcement of hydrogenated carboxylated nitrile–butadiene rubber by multi-walled carbon nanotubes. *Appl. Surf. Sci.* **2008,** *255* (5), 2162-2166.
5. Kueseng, P.; Sae-Oui, P.; Rattanasom, N., Mechanical and electrical properties of natural rubber and nitrile rubber blends filled with multi-wall carbon nanotube: Effect of preparation methods. *Polym. Test.* **2013,** *32* (4), 731-738.
6. 6. Stehr, J., Chemical blowing agents in the rubber industry. Past–present–and future? *Int. Polym. Sci. Technol.* **2016,** *43* (5), 1-10.
7. Yan, N.; Wu, J.; Zhan, Y.; Xia, H., Carbon nanotubes/carbon black synergistic reinforced natural rubber composites. *Plast. Rubber Compos* **2009,** *38* (7), 290-296.
8. Dubey, K.; Bhardwaj, Y.; Rajkumar, K.; Panicker, L.; Chaudhari, C.; Chakraborty, S.; Sabharwal, S., Polychloroprene rubber/ethylene-propylene diene monomer/multiple walled carbon nanotube nanocomposites: synergistic effects of radiation crosslinking and MWNT addition. *J. Polym. Res.* **2012,** *19* (5), 9876.
9. Bizhani, H.; Nayyeri, V.; Katbab, A.; Jalali-Arani, A.; Nazockdast, H., Double percolated MWCNTs loaded PC/SAN nanocomposites as an absorbing electromagnetic shield. *Eur. Polym. J.* **2018,** *100*, 209-218.
10. Wang, G.; Zhao, G.; Wang, S.; Zhang, L.; Park, C. B., Injection-molded microcellular PLA/graphite nanocomposites with dramatically enhanced mechanical and electrical properties for ultra-efficient EMI shielding applications. *J. Mater. Chem. C* **2018,** *6* (25), 6847-6859.
11. Kamal, M. R.; Khoshkava, V., Effect of cellulose nanocrystals (CNC) on rheological and mechanical properties and crystallization behavior of PLA/CNC nanocomposites. *Carbohydr. Polym.* **2015,** *123*, 105-114.
